# The IDO–AhR Axis Controls Th17/Treg Immunity in a Pulmonary Model of Fungal Infection

**DOI:** 10.3389/fimmu.2017.00880

**Published:** 2017-07-24

**Authors:** Eliseu Frank de Araújo, Claudia Feriotti, Nayane Alves de Lima Galdino, Nycolas Willian Preite, Vera Lúcia Garcia Calich, Flávio Vieira Loures

**Affiliations:** ^1^Departamento de Imunologia, Instituto de Ciências Biomédicas, Universidade de São Paulo, São Paulo, Brazil

**Keywords:** paracoccidioidomycosis, indolemine 2,3 dioxygenase, aryl hydrocarbon receptor, Th17 cells, regulatory T cells, innate lymphoid cells

## Abstract

In infectious diseases, the enzyme indoleamine 2,3 dioxygenase-1 (IDO1) that catalyzes the tryptophan (Trp) degradation along the kynurenines (Kyn) pathway has two main functions, the control of pathogen growth by reducing available Trp and immune regulation mediated by the Kyn-mediated expansion of regulatory T (Treg) cells *via* aryl hydrocarbon receptor (AhR). In pulmonary paracoccidioidomycosis (PCM) caused by the dimorphic fungus *Paracoccidioides brasiliensis*, IDO1 was shown to control the disease severity of both resistant and susceptible mice to the infection; however, only in resistant mice, IDO1 is induced by TGF-β signaling that confers a stable tolerogenic phenotype to dendritic cells (DCs). In addition, in pulmonary PCM, the tolerogenic function of plasmacytoid dendritic cells was linked to the IDO1 activity. To further evaluate the function of IDO1 in pulmonary PCM, IDO1-deficient (IDO1^−/−^) C57BL/6 mice were intratracheally infected with *P. brasiliensis* yeasts and the infection analyzed at three postinfection periods regarding several parameters of disease severity and immune response. The fungal loads and tissue pathology of IDO1^−/−^ mice were higher than their wild-type controls resulting in increased mortality rates. The evaluation of innate lymphoid cells showed an upregulated differentiation of the innate lymphoid cell 3 phenotype accompanied by a decreased expansion of ILC1 and NK cells in the lungs of infected IDO1^−/−^ mice. DCs from these mice expressed elevated levels of costimulatory molecules and cytokine IL-6 associated with reduced production of IL-12, TNF-α, IL-1β, TGF-β, and IL-10. This response was concomitant with a marked reduction in AhR production. The absence of IDO1 expression caused an increased influx of activated Th17 cells to the lungs with a simultaneous reduction in Th1 and Treg cells. Accordingly, the suppressive cytokines IL-10, TGF-β, IL-27, and IL-35 appeared in reduced levels in the lungs of IDO1^−/−^ mice. In conclusion, the immunological balance mediated by the axis IDO/AhR is fundamental to determine the balance between Th17/Treg cells and control the severity of pulmonary PCM.

## Introduction

Indoleamine 2,3 dioxygenase-1 (IDO1) is an enzyme that catalyzes the degradation of tryptophan (Trp) along the kynurenines (Kyn) pathway. This enzyme plays a critical role to host defenses against a wide range of pathogens by inducing Trp starvation and controlling inflammatory and immunological reactions. Several types of cells, such as macrophages, epithelial, and dendritic cells (DCs), express IDO1 that can be induced by proinflammatory cytokines (e.g., IFN-γ), toll-like receptor ligands (e.g., lipopolysaccharide), and interactions between immune cells through the engagement of costimulatory molecules (CD80, CD86) on antigen-presenting cells by cytotoxic T-lymphocyte antigen-4 on T cells (1–3). It is known that IDO1 can affect immunity through two non-exclusive mechanisms: (a) establishment of a local response with “amino acid deprivation” that inhibits cell and pathogen proliferation (2); (b) cascade generation of Trp metabolites with specific immunomodulatory or cytotoxic functions that inhibit T cell activation and modulate the differentiation of naïve T cells into regulatory T (Treg) cells (1, 4, 5). Furthermore, Kyn has immunomodulatory effects in the absence of Trp starvation, *via* activation of the transcription factor aryl hydrocarbon receptor (AhR) (6, 7).

Indeed, in fungal infections, IDO1 modulates the mechanisms of host resistance and disease tolerance by inducing Treg cells and inhibiting Th17 differentiation (1, 8–10). Importantly, Treg and Th17 cells share similar developmental pathways (11) and may arise from a common progenitor depending on the balance of inflammatory and anti-inflammatory cytokines (12), presence of retinoic acid (13), and the activation of the AhR, a ligand-activated transcription factor (14, 15).

Paracoccidioidomycosis (PCM), a systemic granulomatous disease caused either by the dimorphic fungi *Paracoccidioides brasiliensis* or *P. lutzii*, is considered the most prevalent deep mycosis in Latin America (16). The inhalation of conidia usually leads to an asymptomatic infection but a few infected individuals evolve to overt disease. The diverse patterns of T cell responses of *P. brasiliensis*-infected individuals are associated with different clinical manifestations. The resistance to infection observed in asymptomatic individuals is mediated by a predominant Th1 response, which is responsible for macrophage activation and fungal killing. The most severe form of the disease, the juvenile form, presents a prevalent Th2/Th9 response and enhanced antibody production. In the chronic inflammatory response characteristic of the adult form of the disease, a prominent Th17 immunity with important participation of Th1 cells was described (17, 18). The polar patterns of the disease could be reproduced in a murine model of pulmonary PCM (19). Resistant mice (A/Sn) develop a chronic regressive disease allied with prevalent Th1/Th17 immunity concomitant with protective CD8^+^ T cells that synthesize large amounts of IFN-γ. The relative protection of susceptible mice (B10.A) is mediated by CD8^+^ T cells that, however, are not able to compensate the CD4^+^ T cell anergy induced by excessive proinflammatory innate response (20–23).

Recent studies of our group have demonstrated that in pulmonary PCM, IDO1 is an important immunoregulatory enzyme that promotes fungal clearance and inhibits T cell immunity and inflammation, with prominent importance to susceptible hosts. Actually, only in the susceptible mouse strain IDO1 inhibition by 1-methyl-dl-tryptophan (1MT) caused progressive tissue pathology and increased mortality rates. In addition, as previously reported in candidiasis and aspergillosis (24, 25), an IDO1-mediated immunomodulatory function of plasmacytoid dendritic cells (pDCs) has been described in pulmonary PCM. The *in vivo* depletion of IDO1 expressing pDCs resulted in less severe disease and increased T cell immunity (26), demonstrating that in pulmonary PCM pDCs have a tolerogenic function as previously described by Pallotta et al. (27). These findings led us to further elucidate the role of IDO1 in pulmonary PCM by comparing the disease developed by IDO1-deficient (IDO1^−/−^) C57BL/6 mice with their normal wild-type (WT) controls. IDO1^−/−^ mice developed a more severe infection with elevated fungal burdens, accompanied by increased inflammatory reactions mediated by prevalent Th17 responses insufficiently controlled by Treg cells. The absence of IDO1 expression has also influenced the expression of AhR and the differentiation of pulmonary innate lymphoid cells (ILCs) by increasing the presence of innate lymphoid cell 3 (ILC3) and reducing NK cells. Both the uncontrolled fungal growth and the exuberant inflammatory reactions appear to have contributed to the exacerbated tissue pathology that resulted in increased mortality rates of IDO1^−/−^ mice.

## Materials and Methods

### Ethics Statement

The experiments were performed in strict accordance with the Brazilian Federal Law 11,794 establishing procedures for the scientific use of animals, and the State Law establishing the Animal Protection Code of the State of São Paulo. All efforts were made to minimize animal suffering. The procedures were approved by the Ethics Committee on Animal Experiments of the Institute of Biomedical Sciences of University of São Paulo (Proc.180/11/CEEA).

### Mice

Eight- to twelve-week-old male C57Bl/6 WT and C57Bl/6 IDO1^−/−^ mice originally obtained from Jackson Laboratories and bred as the specific pathogen-free mice at the Isogenic Breeding Unit of the Department of Immunology, Institute of Biomedical Sciences, University of São Paulo were used throughout this study.

### Fungus and Infection

The highly virulent *P. brasiliensis* 18 isolate (Pb18) was used throughout this study. Yeast cells were maintained by weekly cultivation in Fava Netto culture medium at 36°C and used on days 6–8 of culture. The viability of fungal suspensions, determined by Janus Green B vital dye (Merck), was always higher than 95%. Mice were anesthetized and submitted to intra-tracheal (i.t.) infection as previously described (19). Briefly, after intraperitoneal (i.p.) injection of ketamine and xylazine, animals were infected with 1 × 10^6^ yeast cells, contained in 50 µL of PBS, by surgical i.t. inoculation, which allowed dispensing of the fungal cells directly into the lungs.

### Colony-Forming Unit (CFU) Assays, Mortality Rates, and Histological Analysis

The numbers of viable yeasts in infected organs (lung and liver) were determined by counting the number of CFUs as previously described (28). Mortality studies were done with groups of 10–12 mice. Deaths were registered daily. For histological examinations, the left lung of infected mice was removed and fixed in 10% formalin. Five-micrometer sections were stained by hematoxylin-eosin for an analysis of the lesions and were silver stained (Grocott stain) for fungal evaluation. Morphometrical analysis was performed using a Nikon DXM 1200c camera and Nikon NIS AR 2.30 software. The areas of lesions were measured (in square micrometers) in 10 microscopic fields per slide in 5 mice per group as previously described (29). Results are expressed as the mean ± SEM of total area of lesions for each mouse.

### Assessment of Leukocyte Subpopulations and Flow Cytometric Analysis

The lungs from *P. brasiliensis*-infected WT and IDO1^−/−^ mice were collected after 96 h, 2, and 10 weeks of infection and digested enzymatically for 45 min with collagenase (1 mg/mL; Sigma) in RPMI culture medium (Sigma). Total lung leukocyte numbers were assessed with trypan blue, and viability was always >95%. For cell-surface staining, lung cells were washed and suspended at 1 × 10^6^ cells/mL in staining buffer (PBS, 2% fetal calf serum and 0.1% NaN_3_). Fc receptors were blocked by the addition of unlabeled anti-CD16/32 (eBioscience). The cells were then stained in the dark for 20 min at 4°C with the optimal dilution of each monoclonal antibody. To myeloid cells: anti-CD11b, CD11c, CD40, CD80, CD86, and MHC-II; lymphocytes: CD4, CD25, CD8, CD44, and CD62L; NK: NK1.1, CD49b, NPK46, and Eomes; ILC1: CD127, CD49a, IL-12Rβ1, and Tbet; ILC2: CD127, ICOS, IL-17RB, IL-33, and GATA3; ILC3: CD127, IL-23R, IL-22, and RORC (eBiosciences or BioLegend). Cells were washed twice with staining buffer, fixed with 2% paraformaldehyde (PFA; Sigma). For intracellular detection of cytokines, leukocytes obtained from lungs were stimulated for 6 h in complete RPMI medium containing 50 ng/mL phorbol 12-myristate 13-acetate, 500 ng/mL ionomycin (Sigma), and 3 mM monensin (eBioscience). Next, cells were labeled for surface molecules and then treated according to the manufacturer’s protocol for intracellular staining using the Cytofix/Cytoperm kit (BD Biosciences) and specifics antibodies anti-17, IL-4, IFN-γ, IL, 22, IL-1β, IL-12, TNF-α, IL-6, TGF-β, FoxP3, IDO1, and AhR. Cells were washed twice with staining buffer, suspended in 100 µL, and an equal volume of PFA was added to fix the cells. A minimum of 50,000 events was acquired on FACScanto II flow cytometer (BD Biosciences) using the FACSDiva software (BD Biosciences). Lymphocytes, myeloid cells, NK cells, and ILCs were gated as judged from forward and side light scatter. For Treg cell characterization, FACS plots or histograms were gated on live CD4^+^ CD25^+^ cells and the expression of FoxP3^+^ were determined. The cell-surface expression of leukocyte markers as well as intracellular cytokine expression was analyzed using the FlowJo software (Tree Star).

### RNA Isolation and cDNA Synthesis

Lungs were homogenized in TRIzol reagent using tissue grinders. Phase separation was realized following addition of 0.2 mL chloroform per mL of TRIzol and centrifugation at 12,000 × *g* for 15 min at 4°C. The upper aqueous RNA phase was removed to a new tube and further purified using Ultraclean Tissue and Cells RNA Isolation Kit (MO BIO Laboratories) according to the manufacturer’s protocol. RNA purity and concentration were assessed on a NanoDrop ND-1000 spectrophotometer. An amount of 1 µg total RNA was reverse transcribed in a 20 µL reaction mixture using the High Capacity RNA-to-cDNA kit (Applied Biosystems) following the manufacturer’s directions.

### Real-time Quantitative Polymerase Chain Reaction (RT-PCR)

The cDNA was amplified using TaqMan Universal PCR Master Mix (Applied Biosystems) and pre-developed TaqMan assay primers and probes (*Ahr*, Mm00478932_m1, *Ifng*, Mm001168134_m1, *Tnf*, Mm99999068_m1, *Il-6*, Mm00446190_m1, *Il-10*, Mm00439614_m1, *Tgfb1*, Mm00117882_m1, *Il-17*, Mm00439618_m1, *Il-22*, Mm01226722_m1, *Tbx21*, Mm00450960_m1; *GATA3*, Mm00484683_m1; *Rorc*, Mm01261022_m1; *Foxp3*, Mm00475162_m1; all from Applied Biosystems). PCR assays were performed on an MxP3000P qPCR System and data were developed using the MxPro qPCR software (Stratagene). The average threshold cycle (*C*_T_) values of samples were normalized to *C*_T_ value of *Gapdh* gene. The relative expression was determined by the 2^−ΔΔ^*C*_T_ method.

### Cytokines and Nitric Oxide (NO) Detection

The lungs from *P. brasiliensis*-infected WT and IDO1^−/−^ mice were collected after 96 h, 2, and 10 weeks of infection with one million *P. brasiliensis* yeasts. The organs were aseptically removed and individually disrupted in 7 mL of PBS. Supernatants were separated from cell debris by centrifugation at 3,000 × *g* for 10 min and stored at −80°C. The levels of IL-1β, IL-2, IL-4, IL-6, IL-10, IL-12, IL-17, IL-22, IL-27, IL-35, IL-23, TNF-α, IFN-γ, and TGF-β were measured by capture enzyme-linked immunosorbent assay (ELISA) with antibody pairs purchased from eBioscience or PBL. NO production was quantified by the accumulation of nitrite in the supernatants from *in vitro* protocols by a standard Griess reaction. All determinations were performed in duplicate, and results were expressed as micro molar concentration of NO. Plates were read using a spectrophotometric plate reader (VersaMax, Molecular Devices).

### Statistical Analysis

Differences between groups were analyzed by the Student’s *t*-test or analysis of variance (ANOVA) followed by the Tukey test. For comparisons of greater than two groups, significance was determined using the one- or two-way ANOVA with Tukey’s multiple correction. Differences between survival times were determined with the log-rank test. Calculations were performed using statistical software (GraphPad Prism 7.03). Data are expressed as the mean ± SEM. *p*-Values of ≤0.05 were considered statistically significant.

## Results

### Absence of IDO1 Increases Mortality Rates Associated With Increased Fungal Loads and Tissue Damage

**Figure 1:**
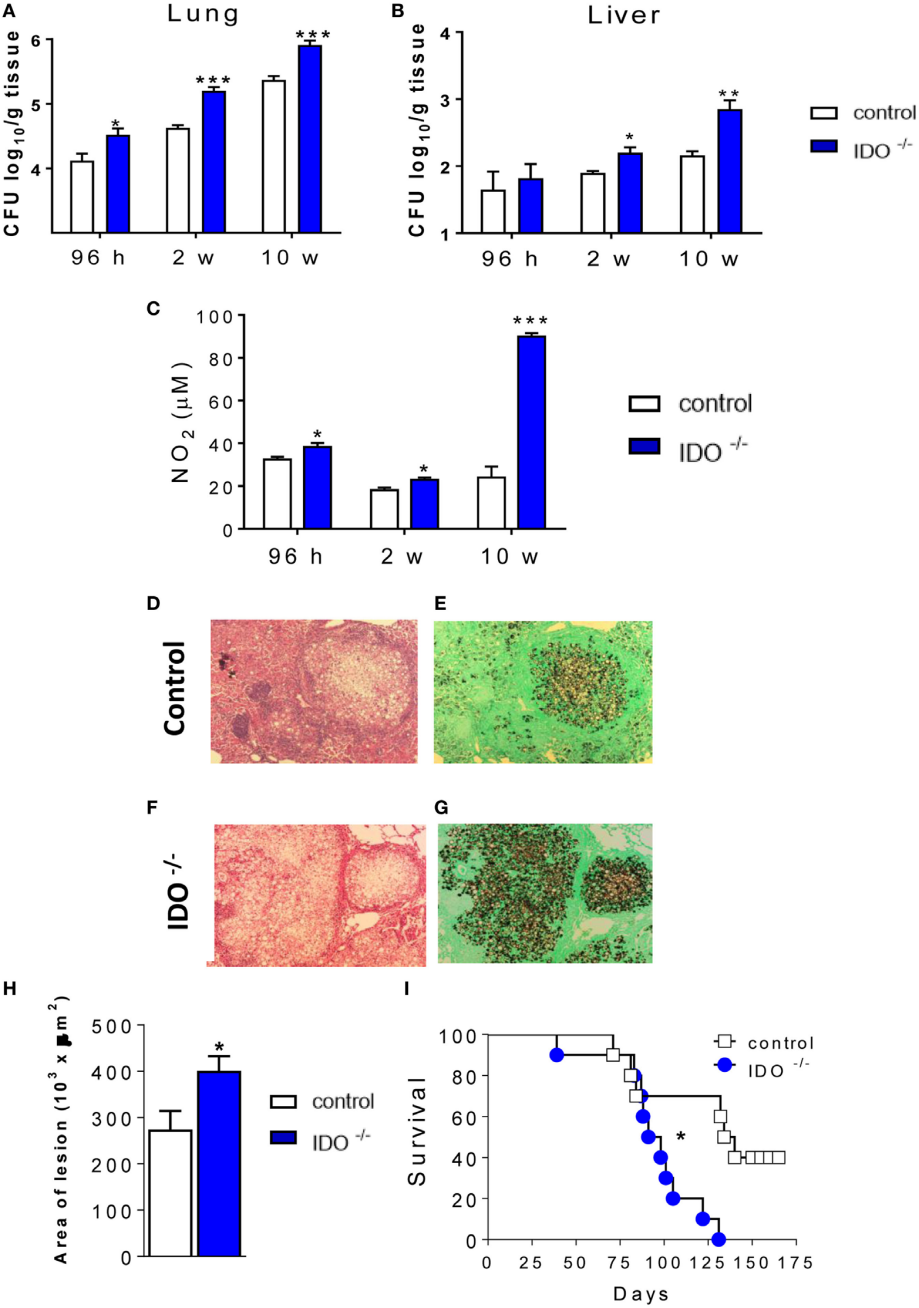
Indoleamine 2,3 dioxygenase-1 (IDO1) controls fungal loads, tissue pathology, and mortality rates. Groups of IDO1^−/−^ and wild-type (WT) C57Bl/6 mice were infected i.t. with 1 × 10^6^ of *Paracoccidioides brasiliensis* yeasts. The colony-forming units (CFUs) from lungs **(A)** and liver **(B)** were determined 96 h, 2, and 8 weeks after infection. The bars represent means ± SEs of the mean (SEM) of log_10_ CFU counts obtained from groups of 4 to 5 mice. **(C)** Supernatants of lung homogenates were obtained from infected mice (*n* = 4–5/time point) and used to determine the levels of nitrite by the Griess reagent. **(D–G)** Photomicrographs of lung lesions of WT control mice **(D,E)** and IDO1^−/−^ mice **(F,G)** at week 10 of infection. Lesions were stained with hematoxylin-eosin (left panels) and Grocott (right panels). **(H)** Morphometrical analysis of lung lesions. The areas of lesions were measured (in square micrometers) in 10 microscopic fields per slide in five mice per group and results expressed as the mean ± SEM of total area of lesions for each mouse. **(I)** Survival curves of WT and IDO1^−/−^ infected mice (*n* = 10) were determined in a period of 175 days. Data represent the means ± SEM and are representative of two independent experiments with equivalent results (*p < 0.05; **p < 0.01, and ***p < 0.001).

C57Bl/6 WT and C57Bl/6 IDO1^−/−^ mice were infected with one million yeasts of *P. brasiliensis* by the i.t. route. After 96 h, 2, and 10 weeks, the severity of fungal infection was assessed by a CFU assay. As shown in the Figure 1, pulmonary (Figure 1A) fungal burdens were increased in IDO1^−/−^ mice at 96 h, 2, and 10 weeks after infection, whereas significant increases in the liver (Figure 1B) after 10 weeks of infection showed that the lung of both IDO1^−/−^ mice were concomitant with increased levels of pulmonary NO (Figure 1C), considered a potent fungicidal mediator in pulmonary PCM. The histopathology analysis (Figures 1D–G) after 10 weeks of infection showed that the lung of both IDO1^−/−^ and WT control mice exhibited large granulomas containing many yeasts and large damaged areas. Comparatively, lung lesions from IDO1^−/−^ were more severe than those of WT mice, involved extensive areas of the lung parenchyma and were composed of isolated or confluent granulomas containing a huge number of fungi (Figures 1F,G). To assess the influence of IDO1 deficiency in the disease outcome, the area of lung lesions and mortality of infected mice were evaluated. The absence of IDO1 expression led to increased lung pathology (Figure 1H) and mortality rates (Figure 1I). At day 127 of infection, all IDO1^−/−^ mice were dead; whereas in the same period, 5 of 10 WT mice were still alive and apparently healthy.

### IDO-1 Expression Increases the Expansion of ILC3 but Reduces ILC1 and NK Cells in the Lungs of *P. brasiliensis*-Infected Mice

**Figure 2:**
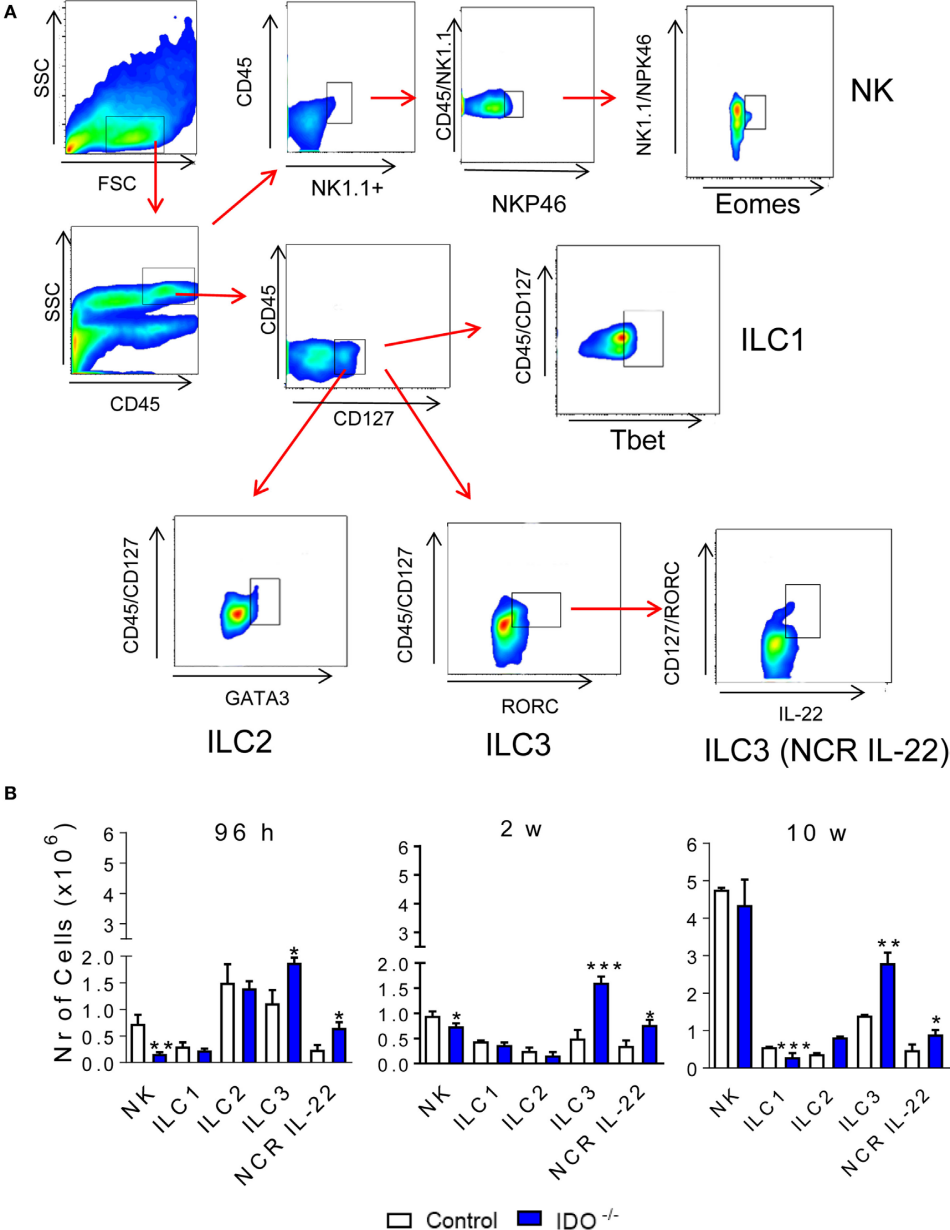
Influence of indoleamine 2,3 dioxygenase-1 (IDO1) expression on the presence of innate lymphoid cells (ILCs) in the lungs of *Paracoccidioides brasiliensis*-infected mice. The phenotypic analysis of ILCs in the lungs of C57Bl/6 wild-type and IDO1^−/−^ mice was performed after 96 h, 2, and 10 weeks after *P. brasiliensis* infection. The lung infiltrating leukocytes were labeled with specific antibodies and analyzed for the phenotypes of NK, ILC1, ILC2, innate lymphoid cell 3 (ILC3), and ILC3 (NCR IL-22) subsets according to the respective markers: for NK: NK1.1, CD49b, NKp46, and Eomes; ILC1: CD127, CD49a, IL-12Rβ1, and Tbet; ILC2: CD127, ICOS, IL-17RB, IL-33, and GATA3; ILC3: CD127, IL-23R, IL-22, and RORC **(A)**. The cell surface and intracellular markers were measured by flow cytometry. One hundred thousand cells were counted and the data expressed by number of positive cells **(B)**. Data are expressed as means ± SEM and are representative of three independent experiments using five mice of each mouse strain per group (*p < 0.05; **p < 0.01, and ***p < 0.001).

Innate lymphoid cells are a new family of lymphocytes that lack specific antigen receptors, produce significant amounts of cytokines, and may be cytotoxic upon activation. The distinct ILC subsets exhibit transcription factors and cytokine signatures found in the CD4^+^ T helper (Th) subpopulations in their responses to specific antigens (30, 31). These features include the shared expression of Tbet and IFN-γ by ILC1 and Th1 cells; GATA-3, IL-5, and IL-13 by Th2 and ILC2 cells; RORC, IL-17, and IL-22 by ILC3 and Th17/Th22 cells, as well as Eomes, IFN-γ, and cytolytic molecules by CD8^+^ T cells and conventional NK cells (30). The previously described influence of IDO/AhR in the differentiation of T and ILC cells (30–33) led us to investigate the influence of IDO1 in the differentiation of pulmonary ILC subsets after *P. brasiliensis* infection of WT and IDO1^−/−^ mice. Therefore, ILC subpopulations were characterized by their expression of specific surface molecules and transcription factors. The NK cells are classified as NK11^+^ CD49b^+^ NKp46^+^ Eomes^+^, the ILC1 are CD127^+^ CD49a^+^ IL-12Rβ1^+^ Tbet^+^, the ILC2 are CD127^+^ ICOS^+^ IL-17RB^+^ IL-33^+^ GATA3^+^, and the ILC3 are CD127^+^ IL-23R^+^ RORC^+^, or the so-called NCR^+^ ILC3^+^, producing IL-22 that are CD127^+^ IL-23R^+^ RORC^+^ IL-22^+^. After 96 h and 2 weeks of infection, a significant decrease in NK cells was detected in the lung infiltrating lymphocytes of IDO1^−/−^ mice. However, at all periods of infection studied, a significant increase in the number of ILC3 and NRC IL-22 was observed in the lungs of IDO1^−/−^ mice. Differences in the influx of ILC1 and ILC2 to the lungs were only observed at week 10 postinfection, when ILC1 appeared in reduced numbers whereas ILC2 appeared in increased numbers in the lungs of IDO1^−/−^ mice (Figure 2).

### IDO1 Controls the Expression of Activation Markers and Intracellular Cytokines of Pulmonary CD11b^+^ and CD11c^+^ Cells

**Figure 3:**
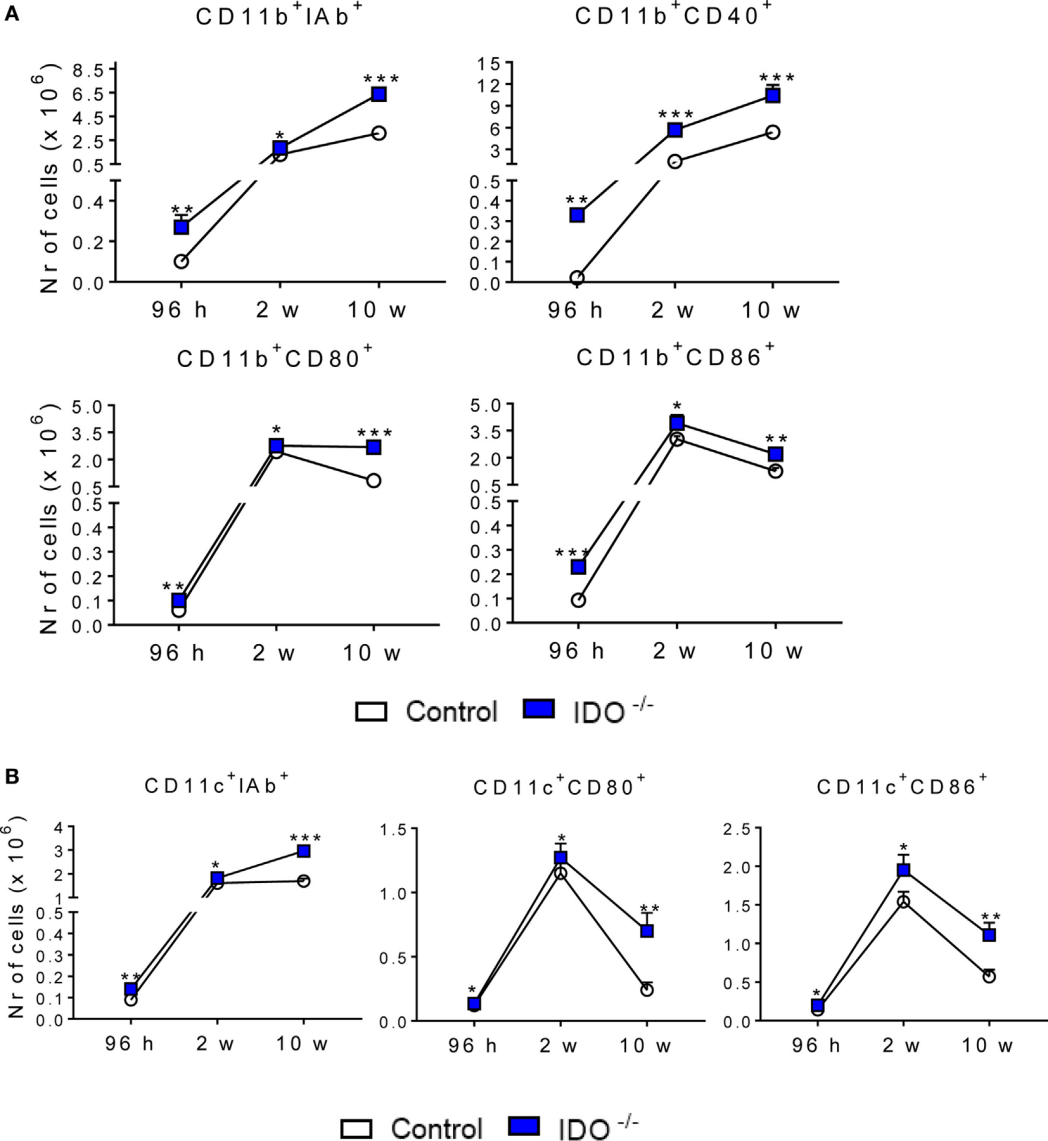
Indoleamine 2,3 dioxygenase-1 (IDO1) controls the expression of activation markers of pulmonary CD11b^+^ and CD11c^+^ cells. The activation markers of CD11b^+^ **(A)** and CD11c^+^ **(B)** leukocytes were measured in lung infiltrating leukocytes of C57Bl/6 wild-type and IDO1^−/−^ after 96 h, 2, and 10 weeks of infection with 1 × 10^6^ *Paracoccidioides brasiliensis* yeasts. The lung cells were obtained as described in Section “Materials and Methods” and labeled with antibodies conjugated to different fluorochromes. The lung infiltrating leukocytes were gated by FSC/SSC analysis. The cells were gated for CD11b^+^ or CD11c^+^ expression and then for the presence of IA^b^, CD40, CD80, and CD86. One hundred thousand cells were acquired on FACS CANTO II and, subsequently, analyzed by FlowJo software. Data are expressed as means ± SEM and are representative of three independent experiments using five mice of each mouse strain per group (*p < 0.05; **p < 0.01, and ***p < 0.001).

IDO1^−/−^ and WT C57BL/6 mice were infected with one million yeasts of *P. brasiliensis* by the i.t. route. After 96 h, 2, and 10 weeks of infection, the lung infiltrating leukocytes were obtained and analyzed for the expression of surface molecules by flow cytometry. We analyzed the expression of some activation molecules (IA^b^, CD40, CD80, and CD86) expressed by CD11b^+^ and CD11c^+^ (Figure 3). When compared with the WT control group, CD11b^+^ (Figure 3A) and CD11c^+^ (Figure 3B) cells from IDO1^−/−^ mice expressed increased levels of IA^b^, CD80, and CD86 at all postinfection periods studied. CD40 was also upregulated in CD11b^+^ cells of IDO1^−/−^ mice.

**Figure 4:**
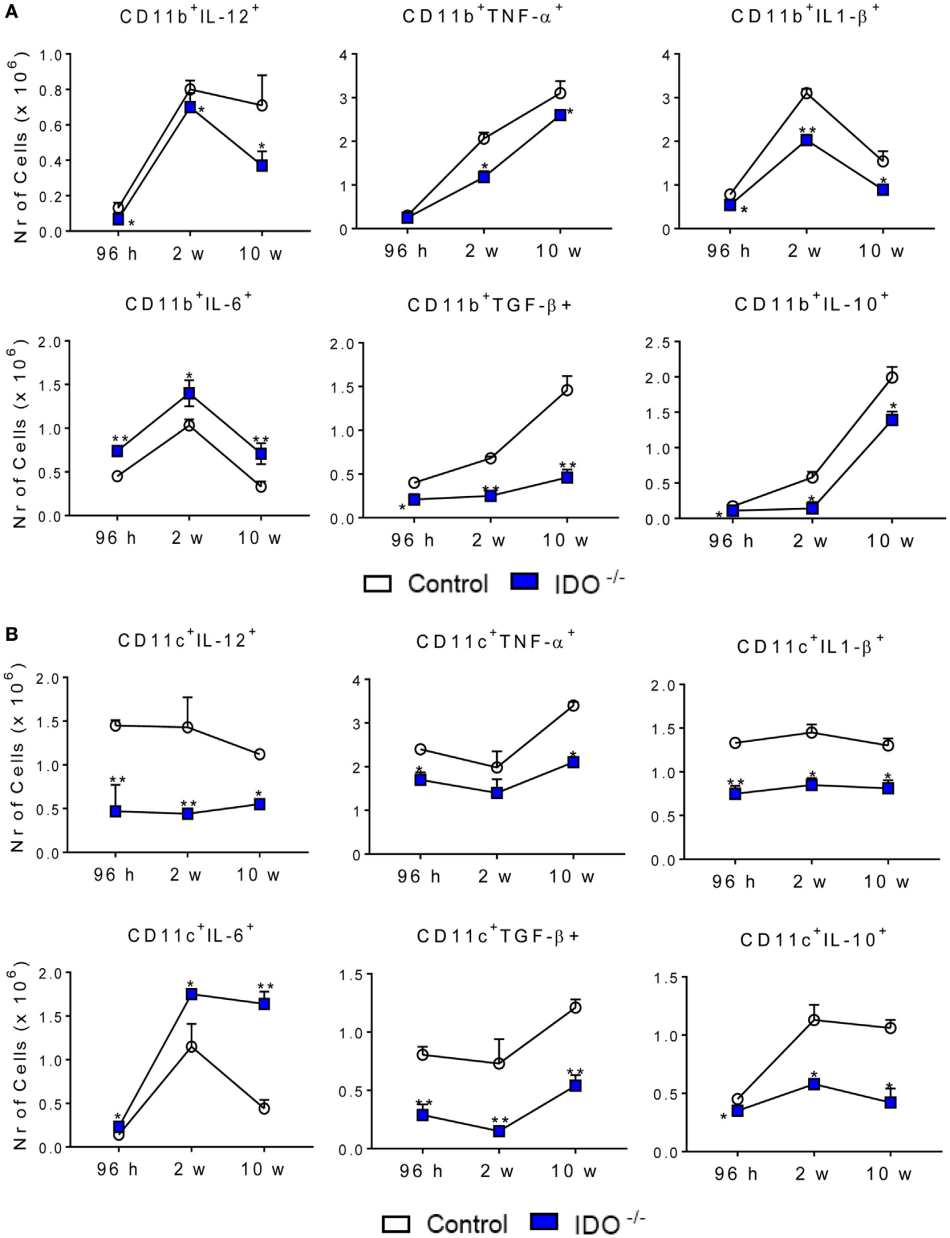
Indoleamine 2,3 dioxygenase-1 (IDO1) expression controls the presence of intracellular cytokines in pulmonary CD11b^+^ and CD11c^+^ cells. The intracellular cytokines were determined in CD11b^+^ **(A)** and CD11c^+^ **(B)** lung infiltrating leukocytes of wild-type and IDO1^−/−^ C57BL/6 mice after 96 h, 2, and 10 weeks of infection with 1 × 10^6^ *Paracoccidioides brasiliensis* yeasts. The lung cells were obtained as described in Section “Materials and Methods” and labeled with antibodies conjugated to different fluorochromes. The lung infiltrating leukocytes were gated by FSC/SSC analysis. The cells were gated for CD11b^+^ or CD11c^+^ expression and then for the presence of cytokines IL-12, TNF-α, IL-1β, IL-10, IL-6, and TGF-β. One hundred thousand cells were acquired on FACS CANTO II and subsequently analyzed by FlowJo software. Data are expressed as means ± SEM and are representative of three independent experiments using five mice of each mouse strain per group (*p < 0.05; **p < 0.01, and ***p < 0.001).

To better define the activation profile of the lung infiltrating leukocytes of both infected mouse strains, the expression of intracellular cytokines (IL-12, TNF-α, IL-1β, IL-6, TGF-β, and IL-10) was characterized by flow cytometry. At all periods after infection analyzed, a decreased number of CD11b^+^ cells expressing intracellular IL-12, TNF-α, IL-1β, TGF-β, and IL-10 was detected in the lungs of IDO1^−/−^ mice when compared to the WT counterparts. By contrast, increased numbers of CD11b^+^ IL-6^+^ cells were found at all postinfection periods in the lungs of IDO^−/−^ mice (Figure 4A). When CD11c^+^ cells were studied, similar results were obtained (Figure 4B), but the differences between IDO1 sufficient and deficient mice was much more evident.

### IDO1 Controls the Expression of AhR in CD11b^+^ and CD11c^+^ Cells

**Figure 5:**
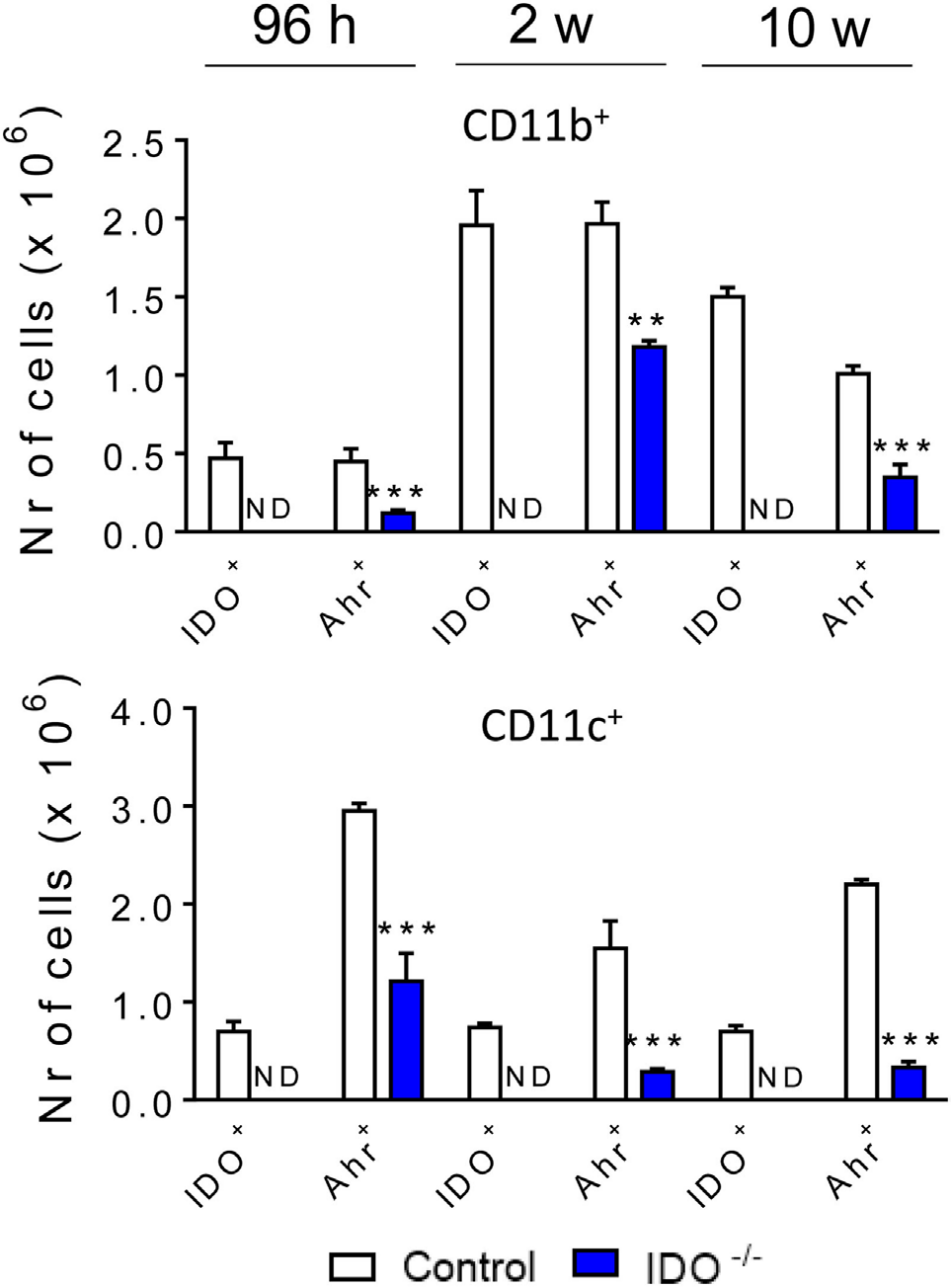
Indoleamine 2,3 dioxygenase-1 (IDO1) expression controls the levels of aryl hydrocarbon receptor (AhR) present in CD11b^+^ e CD11c^+^ cells. The number of IDO1 and AhR expressing cells was determined in lung infiltrating leukocytes of wild-type and IDO1^−/−^ mice 96 h, 2, and 10 weeks after infection with 1 × 10^6^ viable *Paracoccidioides brasiliensis* yeasts. The lung cells were obtained as described in Section “Materials and Methods” and labeled with antibodies conjugated to different fluorochromes. The lung infiltrating leukocytes were gated by FSC/SSC analysis. The cells were gated for CD11b^+^ or CD11c^+^ and then for IDO1 and AhR expression. One hundred thousand cells were acquired on FACS CANTO II and subsequently analyzed by FlowJo software. Data are expressed as means ± SEM and are representative of three independent experiments using five mice of each mouse strain per group (**p < 0.01, and ***p < 0.001).

The influence of IDO1 in the expression of the transcription factor AhR was also investigated in our experimental model. Thus, after 96 h, 2, and 10 weeks of infection with yeasts, the lung infiltrating leukocytes from the IDO1^−/−^ and WT mice were obtained, and the presence of intracellular IDO1 and AhR in CD11b^+^ and CD11c^+^ cells detected by flow cytometry. As depicted in Figure 5, only WT mice expressed intracellular IDO1. The AhR was detected in both mouse strains, but this transcription factor was markedly downregulated in IDO1^−/−^ mice. These results clearly showed that the expression of AhR is dependent on the enzyme IDO1 (Figure 5).

### The Absence of IDO1 Increases the Influx of Naïve and Activated TCD4^+^ and TCD8^+^ Lymphocytes to the Lungs

**Figure 6:**
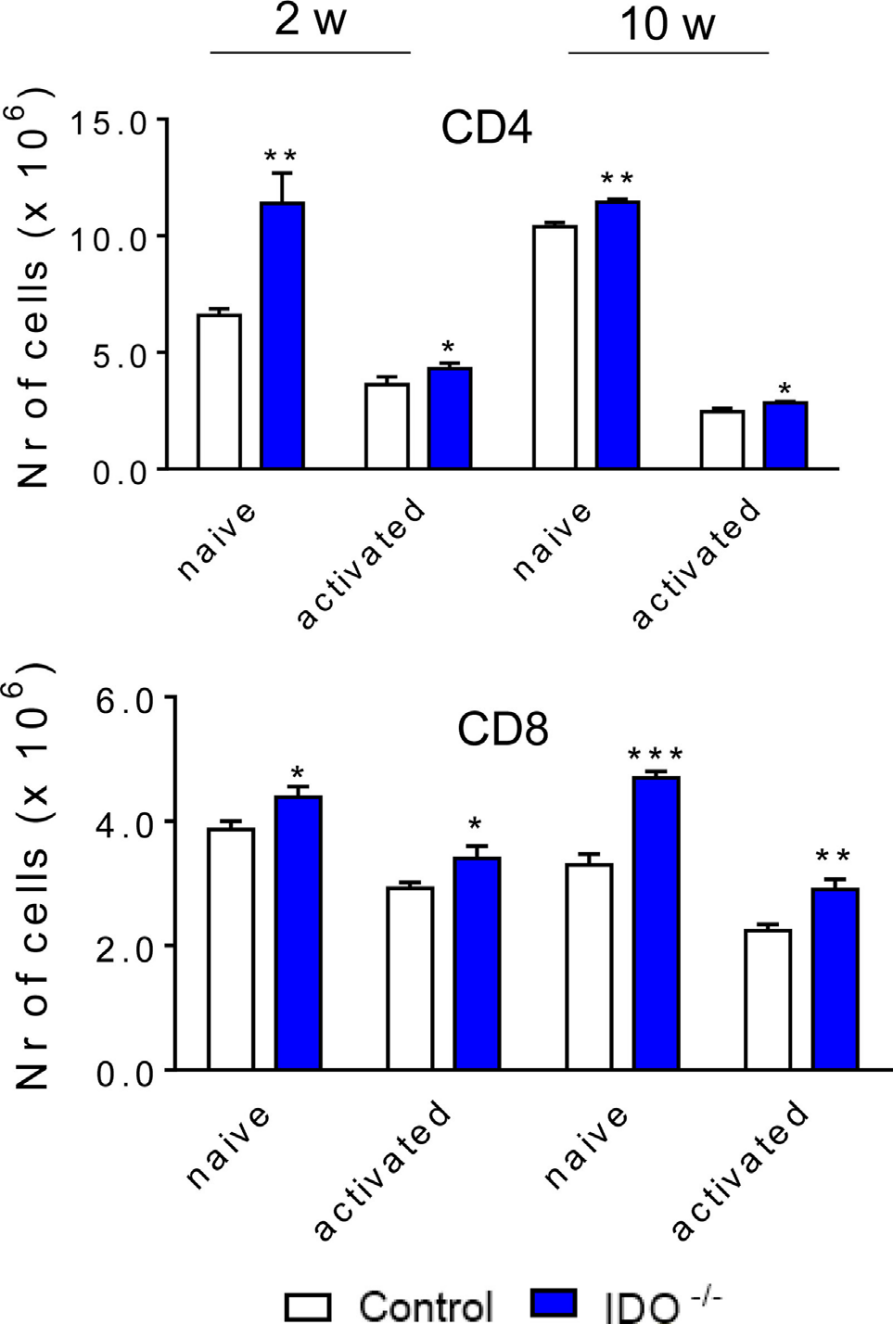
The absence of indoleamine 2,3 dioxygenase-1 (IDO1) induces an increased influx of naïve and activated TCD4^+^ and TCD8^+^ lymphocytes to the lungs. The phenotypic analysis of lung infiltrating lymphocytes from wild-type and IDO1^−/−^ mice was performed at 2 and 10 weeks of *Paracoccidioides brasiliensis* infection. The lung cells were obtained as described in Section “Materials and Methods” and labeled with antibodies conjugated to different fluorochromes. The lung infiltrating leukocytes were gated by FSC/SSC analysis. The cells were then gated for CD4^+^ or CD8^+^ expression and for membrane markers that characterize naïve and effector/activated cells (CD44^low^ CD62L^high^ and CD44^high^ CD62L^low^, respectively). For gate strategy, see Figures S1 and S2 in Supplementary Material. One hundred thousand cells were acquired on FACS CANTO II and subsequently analyzed by FlowJo software. Data are expressed as means ± SEM and are representative of three independent experiments using five mice of each mouse strain per group (*p < 0.05; **p < 0.01, and ***p < 0.001).

We have also assessed whether the absence IDO1 modulates the influx of T cell subpopulations to the lung of *P. brasiliensis*-infected mice. As showed in the Figure 6, after 2 and 10 weeks of infection, a higher number of both naive (CD4^+^ CD44^low^ CD62L^high^) and effector/activated (CD4^+^ CD44^high^ CD62L^low^) CD4^+^ T cells were found in the lungs of IDO1^−/−^ mice when compared with WT counterparts. Similarly, a larger number of naïve and activated CD8^+^ T cells (CD8^+^ CD44^low^ CD62L^high^ and CD8^+^ CD44^high^ CD62L^low^, respectively) were detected in the lungs of IDO1^−/−^ mice when compared with WT controls (Figure 6).

### IDO1 Controls the Secretion of Pulmonary Cytokines

**Figure 7:**
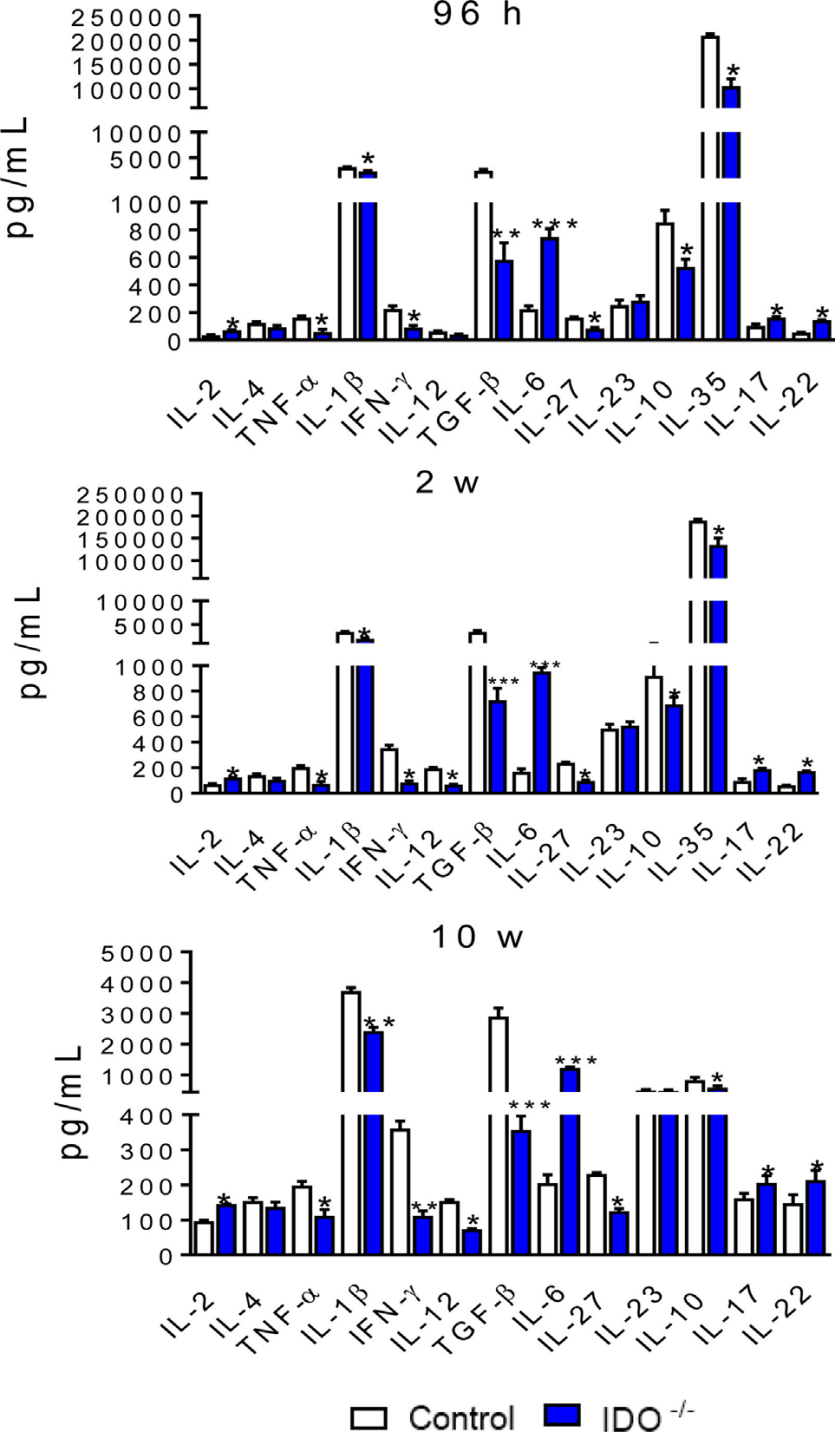
Indoleamine 2,3 dioxygenase-1 (IDO1) regulates the levels of pulmonary cytokines. Quantitation of cytokines in lung homogenates of wild-type and IDO1^−/−^ mice infected with 1 × 10^6^ viable *Paracoccidioides brasiliensis* yeasts. The levels of cytokines were measured by enzyme-linked immunosorbent assay in lung homogenates obtained at 96 h, 2, and 10 weeks of infection. Bars show mean ± SEM from at least four mice per group and are representative of two independent experiments (*p < 0.05, **p < 0.01, and ***p < 0.001).

Lung homogenates were obtained from infected IDO1^−/−^ and control WT mice at 96 h, 2, and 10 weeks of infection. Cytokines were measured by ELISA. Similar patterns of cytokines production were seen at all postinfection periods studied. Most cytokines (TNF-α, IL-1β, IFN-γ, TGF-β, IL-27, IL-10, and IL-35) appeared in decreased levels, whereas IL-2, IL-6, IL-17, and IL-22 were detected in higher concentrations than those detected in WT mice (Figure 7).

### IDO1 Influences the Expression of Cytokines and Transcription Factors Genes

**Figure 8:**
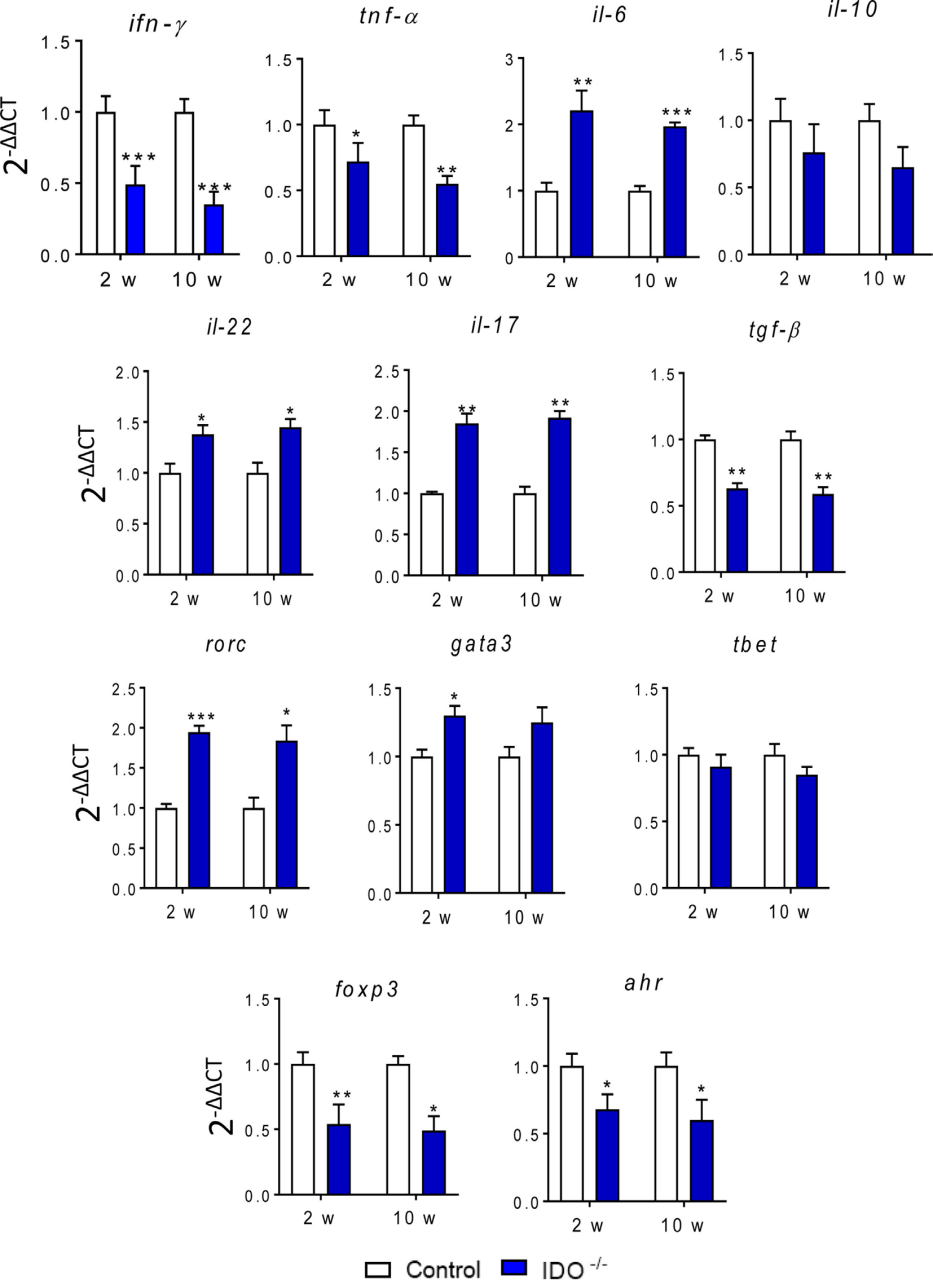
Indoleamine 2,3 dioxygenase-1 (IDO1) influences the gene expression of cytokines and transcription factors. Relative expression of mRNA for aryl hydrocarbon receptor (AhR), IFN-γ, TNF-α, IL-6, RORC, Tbet, GATA3, FoxP3, IL-10, TGF-β, IL-17, and IL-22 in whole lung cells of wild-type and IDO1^−/−^ mice after 10 weeks of *Paracoccidioides brasiliensis* infection. The level of gene transcription was determined by real-time PCR. Bars show mean ± SEM from at least four mice per group and are representative of three independent experiments (*p < 0.05; **p < 0.01, and ***p < 0.001).

We have also assayed the gene expression of transcription factors and cytokines involved in Th1, Th2, Th17, Th22, and Treg differentiation as well as the transcription of AhR gene associated with IDO1 activity. Thus, the mRNA expression in whole lung cells of WT and IDO1^−/−^ mice was determined at weeks 2 and 10 after infection by RT-PCR using appropriate primers. As shown in Figure 8, the absence of the IDO1 led to a marked decrease in the relative expression of IFN-γ mRNA, a cytokine associated with the induction and catalytic function of the enzyme. The mRNA expression for IFN-γ, TNF-α, TGF-β, and IL-10 also appeared in decreased levels, whereas IL-6, IL-17 and IL-22 mRNAs were upregulated in IDO1^−/−^ mice. In both postinfection periods, a concomitant decrease in mRNA for AhR and Foxp3 transcription factors were observed in IDO1 deficient mice. By contrast, RORC and GATA3 mRNAs were significantly upregulated IDO1^−/−^ mice, but minor differences were seen in Tbet expression.

### The Absence of IDO1 Increases the Number of Th17 but Reduces the Presence of Th1 and Treg Cells

**Figure 9:**
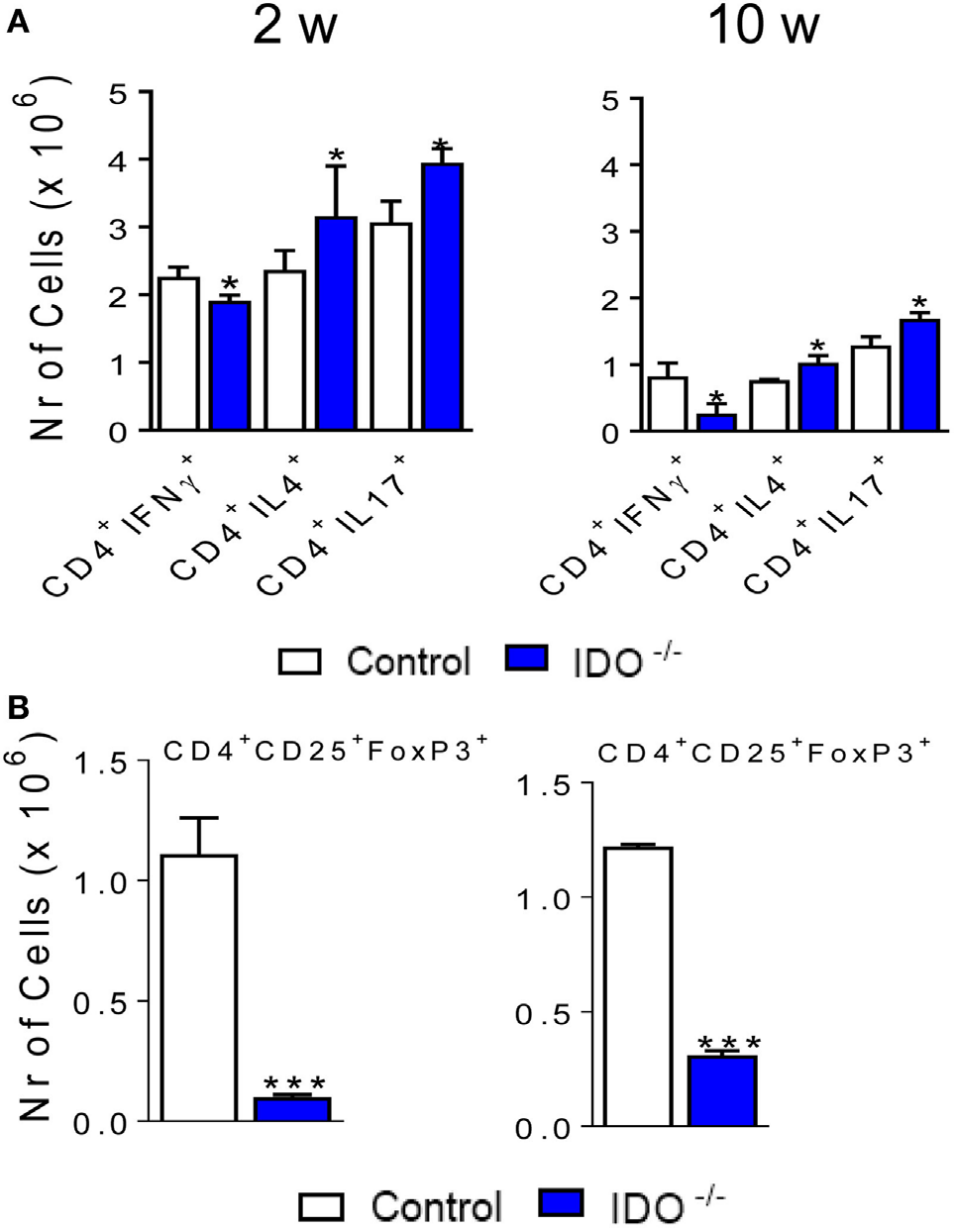
The absence of indoleamine 2,3 dioxygenase-1 (IDO1) increases the number of pulmonary Th17 but reduces the presence of Th1 and regulatory T (Treg) cells. The phenotypic analysis of lung infiltrating lymphocytes from wild-type and IDO1^−/−^ mice was performed at 2 and 10 weeks of *Paracoccidioides brasiliensis* infection. The lung cells were obtained as described in Section “Materials and Methods” and labeled with antibodies conjugated to different fluorochromes. The lung infiltrating leukocytes were gated by FSC/SSC analysis. **(A)** The cells were gated for CD4^+^ and then for intracellular expression of IL-4, IL-17, IL-22, or IFN-γ. **(B)** For Treg cells characterization, the FoxP3^+^ cells were counted on CD4^+^ CD25^+^ double-positive cells. For gate strategy, see Figure S3 in Supplementary Material. One hundred thousand cells were acquired on FACS CANTO II and subsequently analyzed by FlowJo software. Data are expressed as means ± SEM and are representative of three independent experiments using five mice of each mouse strain per group (*p < 0.05 and ***p < 0.001).

It has been demonstrated in fungal infections the fundamental role of IDO1 in promoting the balance between T cell subpopulations (8, 9). IDO1 and Kyn, have the ability of inducing Treg and inhibit Th17 cells, contributing to the regulatory mechanisms that govern immunity or tolerance to pathogens (1, 2, 10). Therefore, we have also characterized the Th subpopulations in the course of *P. brasiliensis* infection of IDO1-deficient and -sufficient mice. As depicted in Figure 9A, the IDO1 enzyme consistently regulates the differentiation of Th lymphocytes. In the absence of IDO1, decreased numbers of CD4^+^ IFN-γ^+^ T cells, which define the Th1 subset, were found. Regarding the Th2 subpopulation, only at the 10th week postinfection, a significant increase in the number of CD4^+^ IL-4^+^ cells were detected in the lungs of IDO1^−/−^ mice. After 2 and 10 weeks of infection, CD4^+^ IL-17^+^ cells appeared in higher numbers in the lungs of IDO1^−/−^ mice when compared with their WT controls. Furthermore, a marked reduction in pulmonary Treg (CD4^+^ CD25^+^ Foxp3^+^) cells was observed in IDO1^−/−^ mice at weeks 2 and 10 of infection (Figure 9B).

## Discussion

The characterization of the infectious environment is particularly important to define how the innate immunity influences the subsequent development of adaptive immunity. The regulatory mechanisms that operate at both phases of immunity are also fundamental to determine how the immune response will dictate the severity of an infectious process. In this context, we have explored the regulatory mechanism exerted by IDO1 in both phases of immunity against *P. brasiliensis* infection. It was quite interesting to verify the close association between T cell phenotypes and those of ILCs, a class of innate cells present at the site of infection at the early phase of infection that plays an important role in the further definition of adaptive immunity (34). We could demonstrate for the first time that ILC participate in the immune response against pulmonary PCM. The expansion of these ILCs was influenced by the IDO1 activity that controls the initial cytokines microenvironment and the induction of transcription factors that determine ILC and T cell phenotypes. Reduced Eomes and Tbet led, respectively, to diminished NKp46 and ILC1, whereas increased RORC resulted in increased ILC3, including those associated with increased production of IL-22, despite the reduced AhR mRNA and protein, which were previously shown to be involved in the synthesis of IL-22 (15).

Previous studies demonstrated that IDO1 plays an important role in the immunity against commensal fungi such as *Aspergillus fumigatus* and *Candida albicans* by regulating fungal tolerance mediated by prevalent activity of IDO and Treg cells and fungal immunity mediated by Th1/Th17 cells properly controlled by Treg cells (24, 35, 36). Here, we demonstrated that in pulmonary PCM, IDO1 controls both, fungal burdens and inflammatory reactions, functions that were concomitant with the expression of AhR. The absence of IDO1 expression increases fungal burdens potentiating the expression of activation markers in macrophages and DCs resulting in exacerbated Th17 immunity poorly controlled by Treg cells. This is the worst scenario for a host response where the uncontrolled fungal growth is accompanied by intense but inefficient inflammatory reactions causing exaggerated tissue pathology that ultimately leads to the precocious death of infected mice.

In vaginal candidiasis, the selective deficiency of IDO1 significantly increased fungal loads and tissue damage mediated by inflammatory cells (37) and here a similar result was observed. Our finding of elevated fungal loads may be attributed to the inability of IDO1^−/−^ mice to deplete Trp, which possibly contributed to the enhanced *P. brasiliensis* growth. However, the more severe infection of IDO1^−/−^ mice was accompanied by increased levels of NO, a well-recognized fungicidal mediator in *P. brasiliensis* infection (38–44). Actually, a dual role regarding NO activity has been observed in PCM. It can enhance the killing ability of IFN-γ activated macrophages exerting a protective effect (40–42) but is also suppressive as previously demonstrated (39, 43, 44). Interestingly, Bernardino et al. using iNOS^−/−^ mice clearly showed that absence of NO production is accompanied by an early reduction in fungal loads and increased T cell responses. At the chronic phase of the disease, iNOS^−/−^ mice showed increased numbers of viable yeasts circumscribed, however, by elevated presence of activated macrophages and T cells (39). Importantly, the inducible isoform of nitric oxide synthase (iNOS) responsible for NO production and the enzyme IDO1 are induced by IFN-γ (45, 46), but NO and IDO1 are mutually inhibitory (47–49). This reciprocal regulation was here detected, since the absence of IDO1 expression enhanced NO production that, however, was insufficient to control the fungal growth. These results were consistent with our previous studies in resistant and susceptible mice where increased NO synthesis paralleled the IDO1 inhibition by 1MT (50). More than that, these results demonstrate that, contrary to the well accepted paradigm, in murine PCM the control of fungal growth by IDO1 activity is more relevant than that mediated by NO production.

At all postinfection periods analyzed, cells from IDO1^−/−^ mice showed increased levels of activation molecules that can explain the increased expansion of T cell that migrate to the lungs of IDO1^−/−^ mice. Despite its statistical significance, it is difficult to point out the biological relevance of the increased expression of costimulatory molecules by DC11b^+^ cells. However, our previous report (26) showed that CD11c^+^ cells from IDO^−/−^ mice have an enhanced ability of inducing T cell proliferation. DCs from IDO^−/−^ mice have an increased ability of expanding CD4^+^ and CD8^+^ T lymphocytes in parallel with decreased proliferation of Treg cells (CD4^+^ CD25^+^ Foxp3^+^ T cells). This study has also shown that the CD4^+^ and CD8^+^ T cells exhibited an activated phenotype as revealed by the expression of CD25 and DC69. Interestingly, both CD11b^+^ and CD11c^+^ cells showed significantly reduced levels of pro- and anti-inflammatory cytokines, in parallel with increased presence of IL-6^+^ cells. These effects were more prominent in CD11c^+^ than in CD11b^+^ cells as expected by the major expression and activity of IDO1 by the former leukocyte subpopulation (2, 51). Furthermore, the new cytokines balance in CD11b^+^ and CD11c^+^ cells of IDO1^−/−^ mice led to increased proliferation of ILC3 and Th17 lymphocytes that are known to be expanded by IL-6 and TGF-β signaling, STAT3 phosphorylation, and expression of RORγτ, the molecular signature of IL-17 secreting cells.

It has been reported that IDO1 and AhR expression are mutually regulated (1, 52), and here we could demonstrate the profound impairment of AhR production caused by IDO1 deficiency. These findings can explain the increased inflammatory reactions developed by IDO1^−/−^ mice and the phenotype of T cells and ILC developed by *P. brasiliensis*-infected IDO1^−/−^ mice: increased presence of ILC3 and Th17 cells associated with reduced numbers of Treg cells.

The elevated numbers of naïve and activated lymphocytes found in the lung of IDO1^−/−^ mice were consistent with the higher levels of IL-2 produced by IDO1^−/−^ mice, a fact also observed in our studies with IDO1 inhibition by 1MT (50). In addition, the decreased levels of IL-12, TNF-α, and IFN-γ reflect the reduced expansion of Th1 cells as shown by the low numbers of CD4^+^ IFN-γ^+^ lymphocytes present in the lungs of IDO^−/−^ mice. The reduced levels of IL-10, TGF-β, and IL-35 are also concordant with the drastic reduction in the expansion and migration of Treg cells to the lungs of IDO1^−/−^ mice. Furthermore, the blockade of the IDO/AhR axis increased the levels of IL-17 and IL-22 secreted into the lungs parenchyma and reflected the major development of Th17 cells that have the ability of secreting both cytokines. The same shift for the IL-17/IL-22 production was reported by De Luca et al. (37) studying the effect of genetic IDO1 deficiency in vaginal candidiasis.

It is apparently conflicting the elevated levels of IL-22 and reduced presence of AhR observed in the lungs of IDO1^−/−^-infected mice. IL-22 belongs to the IL-10 family of cytokines, is produced by many cell subpopulations, including Th22, Th1, and Th17, classical and non-classical NK cells (NK-22), NKT cells, and ILC3 (15, 53–58). IL-22 plays an important role in fungal infections due to its ability of epithelial tissue repair and the induction of antimicrobial peptides (37, 59–61). Several transcription factors, including STAT3, RORC, and AhR, have been described as essential regulators of IL-22 (15) and AhR^−/−^ mice are highly compromised in terms of IL-22 production (15, 62). By inducing IL-22-producing ILC, AhR can promote initial fungal clearance, and by expanding Treg cells contributes with disease tolerance but both mechanisms cooperate to maintain host homeostasis (37). The increase in IL-22 here observed could not be ascribed to the effect of AhR on T cells but to the increased influx of IL-22-secreting Th17 and ILC3 into the lungs of IDO1^−/−^ mice. Indeed, besides Th17 cells, an increased presence of ILC3 positive for intracellular IL-22 were seen in the whole course of infection of IDO1^−/−^ mice.

The increased number of effector T cells in the lung cell infiltrates of IDO1-deficient mice was concurrent with a vigorous reduction of CD4^+^ CD25^+^ FoxP3^+^ Treg cells. This decreased expansion can be attributed to the impaired production of Kyn and the reduced activity of AhR, a transcription factor that needs its ligand to upregulate the transcription of FoxP3 necessary to Treg cells differentiation (63, 64). Indeed, the IDO1 enzyme and its catalytic products can act as a bridge between DCs and Treg cells that can use reverse signaling and non-canonical NFκB activation of DCs for its suppressive effector function and self-propagation (3). The low numbers of Treg cells were concomitant with the reduced levels of several suppressive cytokines detected in the lung cell infiltrates of IDO1^−/−^ mice. IL-27 has a wide range of immunomodulatory activities. Although it may promote the development of Th1, IL-27 can suppress T cell responses and the development of pathogenic Th17 cells (65, 66), functions that were possibly impaired in the immune response developed by IDO1^−/−^ mice. Besides IL-27, the low levels of typical Treg cytokines (TGF-β, IL-35, and IL-10) possibly contributed to the enhanced Th17 inflammatory reactions developed by IDO1-deficient mice.

The IDO1 activity has been associated with the tolerogenic function of plasmacytoid DCs (26), a phenomenon also described in our pulmonary model of PCM. We verified that *P. brasiliensis*-infected pDCs enhanced the expansion of Treg cells and reduce the expansion and activation of Th1 and Th17 cells. This finding was not detected when pDCs from IDO1^−/−^ mice were used. More importantly, the *in vivo* depletion of pDC by a specific antibody resulted in reduced levels of IDO1 and regulatory cytokines in the lungs of pDC-depleted mice, once more highlighting the close connection between IDO1 and expression of immunosuppressive cytokines in PCM (26).

Although no studies have directly addressed the role of IL-17 in PCM, several indirect findings have clearly demonstrated that the well-balanced production of pro- and anti-inflammatory cytokines is crucial for establishing protective immunity. Indeed, the absence of TLR2 signaling induces an excessive tissue pathology associated with increased Th17 differentiation and impaired Treg development (67). By contrast, in the case of TLR4 deficiency, the excessive proliferation of Treg cells and reduced Th17 differentiation led to more severe disease due to uncontrolled fungal burden (68). In addition, the immunity mediated by the adoptive transfer of effector T cells without the concomitant presence of Treg cells led to increased Th17 differentiation and tissue pathology (69). These previous findings and those reported here clearly indicate that the balanced differentiation of Th1/Th17/Treg cells is fundamental for achieving protection in pulmonary PCM.

In conclusion, the IDO1–Ahr–Treg axis was strongly impaired in the absence of the IDO1 expression clearly demonstrating that this regulatory loop plays an important role in the control of immunity and severity of pulmonary PCM.

## Ethics Statement

The experiments were performed in strict accordance with the Brazilian Federal Law 11,794 establishing procedures for the scientific use of animals, and the State Law establishing the Animal Protection Code of the State of São Paulo. All efforts were made to minimize animal suffering. The procedures were approved by the Ethics Committee on Animal Experiments of the Institute of Biomedical Sciences of University of São Paulo (Proc.180/11/CEEA).

## Author Contributions

Conceived and designed experiments: EA, VC, and FL. Contributed with reagent: FL and VC. Performed the experiments: EA, CF, NG, NP, and FL. Analyzed the data: CF, VC, FL, and EA. Wrote the paper: EA, NP, VC, and FL.

## Conflict of Interest Statement

The authors declare that the research was conducted in the absence of any commercial or financial relationships that could be construed as a potential conflict of interest.
