# Supplementary figures and images for: The IDO–AhR Axis Controls Th17/Treg Immunity in a Pulmonary Model of Fungal Infection
Source: Front Immunol. 2017 Jul 24;8:880. doi: 10.3389/fimmu.2017.00880 (PMC5523665; doi:10.3389/fimmu.2017.00880)

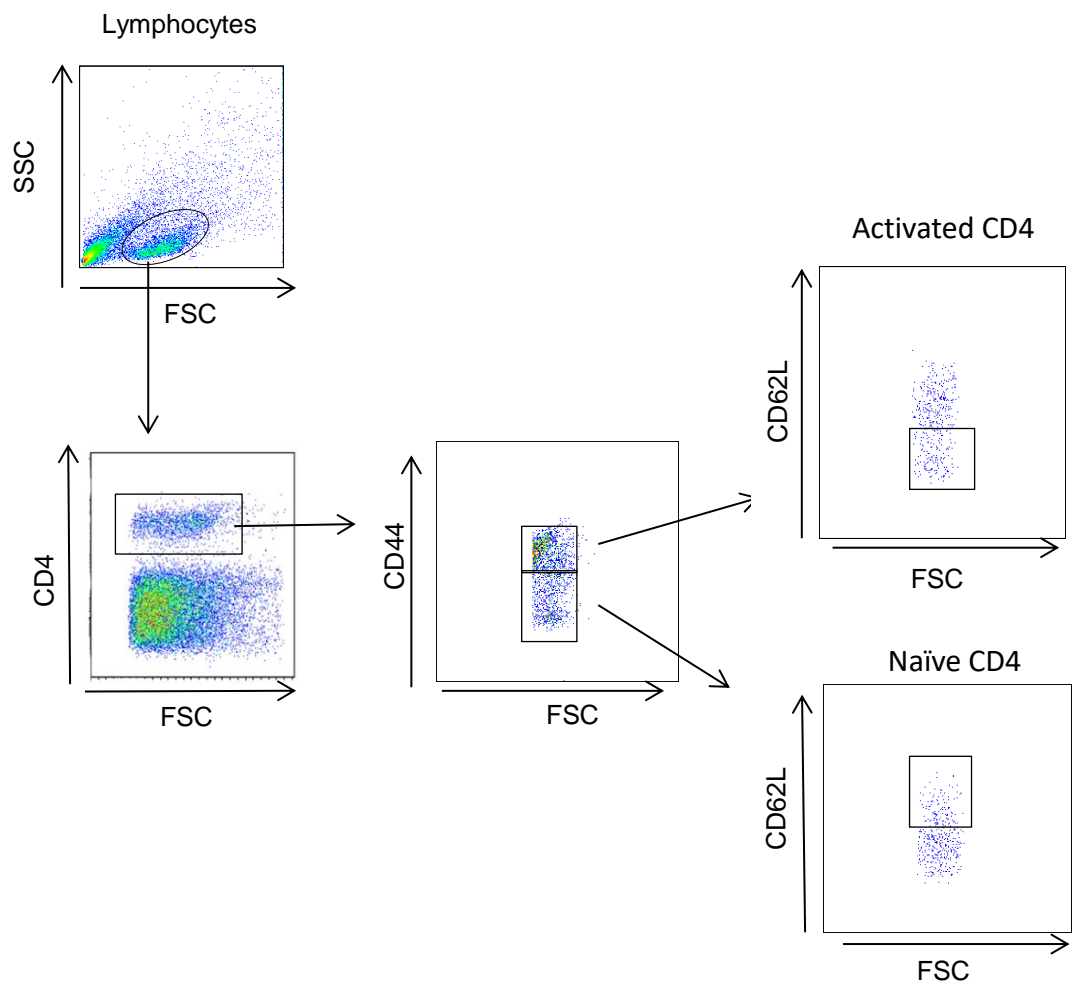

Supplement: Supplementary file 1 [file Image_1.PDF]

# Lymphocytes

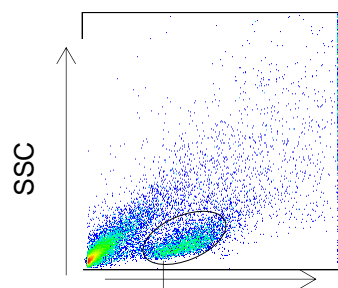

FSC

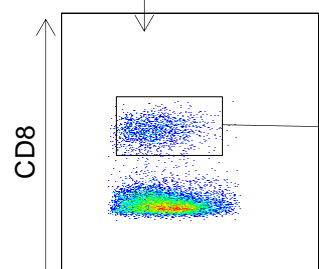

CD8

FSC

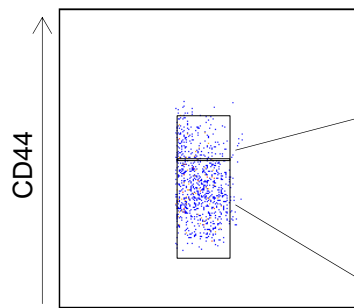

CD44

FSC

## Activated CD8

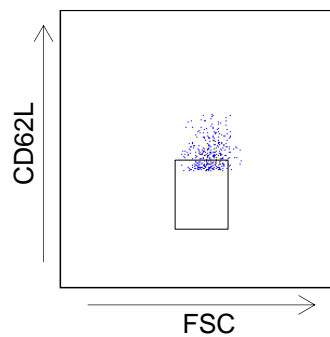

CD62L

FSC

## Naïve CD8

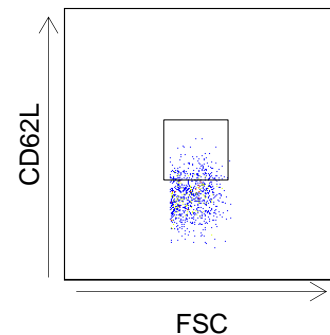

CD62L

FSC

Supplement: Supplementary file 2 [file Image_2.PDF]

# Lymphocytes

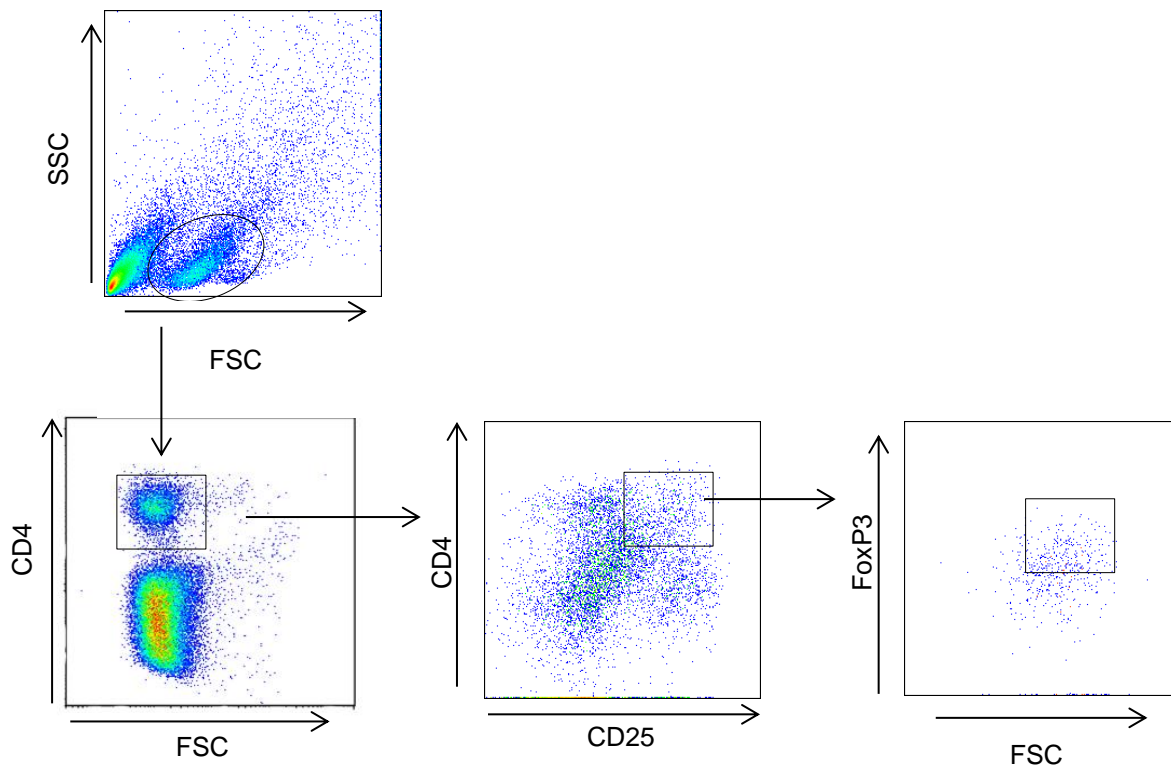

# Lymphocytes

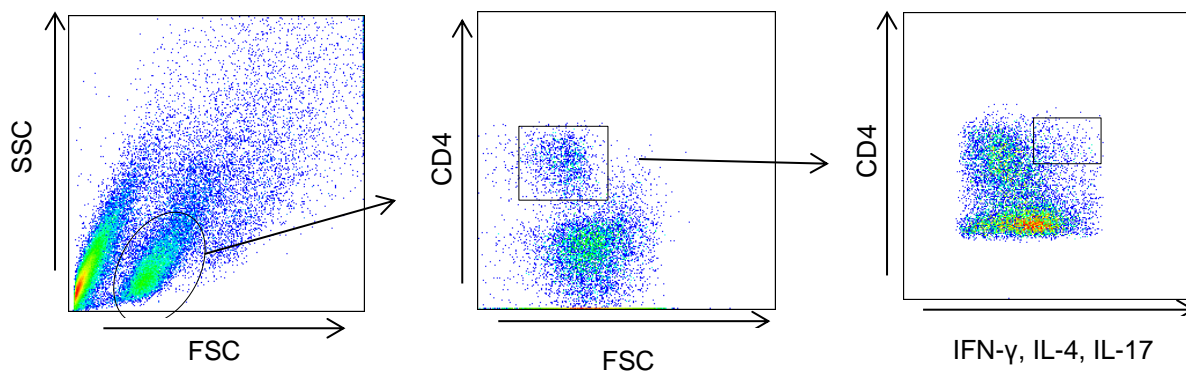

Supplement: Supplementary file 3 [file Image_3.PDF]
